# Supplementary material for: Shedding Light on Osteosarcoma Cell Differentiation: Impact on Biomineralization and Mitochondria Morphology
Source: Int J Mol Sci. 2023 May 10;24(10):8559. doi: 10.3390/ijms24108559 (PMC10218373; doi:10.3390/ijms24108559)
Supplement: Supplementary file 1 [file ijms-24-08559-s001.zip › ijms-2386357-supplementary.pdf]

Supplementary information

The impact of osteosarcoma cell differentiation on biomineralization: lights and shadows on the mitochondrial involvement in hydroxyapatite formation

Francesca Rossi [1, †], Giovanna Picone [1, †], Concettina Cappadone [1], Andrea Sorrentino [2], Marta Columbaro [3], Giovanna Farruggia [1,4], Emilio Catelli [5], Giorgia Sciutto [5], Silvia Prati [5], Robert Oliete [2], Alice Pasini [6], Eva Pereiro [2], Stefano Iotti [1,4, ‡] and Emil Malucelli [1, *, ‡]

1 Department of Pharmacy and Biotechnology, University of Bologna, 33-40126 Bologna, Italy

2 Mistral Beamline, ALBA Synchrotron Light Source, Cerdanyola del Valles, 08290 Barcelona, Spain

3 Piattaforma di Microscopia Elettronica, IRCCS Istituto Ortopedico Rizzoli, 40136 Bologna, Italy

4 National Institute of Biostructures and Biosystems (NIBB), 00136 Rome, Italy

5 Department of Chemistry "G. Ciamician", Università di Bologna, via Selmi 2, I-40126 Bologna, Italy

6 Department of Electrical, Electronic and Information Engineering "Guglielmo Marconi" (DEI), University of Bologna, via dell'Università 50, 47522 Cesena, Italy

* Emil Malucelli: emil.malucelli@unibo.it

† These authors equally contributed to this work.

‡ These authors share senior authorship.

Video S1: Cryo-XANES spectro-microscopy carried out on treated SaOS-2 cells after 4 days of osteogenic treatment. Average of absorbance projections recorded at the pre-Ca-edge energy region and at the Ca $L_{2,3}$ peak maxima (L3 ≈ 349.2 eV; L2 ≈ 352.9 eV) highlights the presence of Calcium containing structures inside round mitochondria.

Video S2: Tomography of a round mitochondrion and corresponding 3D reconstruction: Ca structures are highlighted in pink and mitochondrion external membrane is in light-blue.

Video S3: Tomography of a 10 D treated sample acquired at 352.9 eV highlights mitochondria containing highly absorbing structures not made of Ca, mitochondria during a fusion process and mitochondrial network.
